# Visualization using NIPTviewer support the clinical interpretation of noninvasive prenatal testing results

**DOI:** 10.1186/s12920-025-02086-8

**Published:** 2025-01-20

**Authors:** Patrik Smeds, Izabella Baranowska Körberg, Malin Melin, Claes Ladenvall

**Affiliations:** 1 Department of Immunology, Genetics and Pathology, Rudbeck Laboratory, Uppsala University, Uppsala, SE-751 85 Sweden; 2 Clinical Genomics Uppsala, Science for Life Laboratory, Uppsala University, Uppsala, Sweden; 3 Department of Clinical Genetics, Uppsala University Hospital, Uppsala, Sweden

**Keywords:** Non-invasive prenatal testing (NIPT), Diagnostics, Cell-free DNA, Screening, Aneuploidy, Trisomy, VeriSeq NIPT solution, Web application

## Abstract

**Background:**

Noninvasive prenatal testing (NIPT) is increasingly used to screen for fetal chromosomal aneuploidy by analyzing cell-free DNA (cfDNA) in peripheral maternal blood. The method provides an opportunity for early detection of large genetic abnormalities without an increased risk of miscarriage due to invasive procedures. Commercial applications for use at clinical laboratories often take advantage of DNA sequencing technologies and include the bioinformatic workup of the sequence data. The interpretation of the test results and the clinical report writing, however, remains the responsibility of the diagnostic laboratory. In order to facilitate this step, we developed NIPTviewer, a web-based application to visualize and guide the interpretation of NIPT data results.

**Results:**

NIPTviewer has a database functionality to store the NIPT results and a web interface for user interaction and visualization. The application has been implemented as part of a novel analysis pipeline for NIPT in a diagnostic laboratory at Uppsala University Hospital. The validation data set included 84 previously analyzed plasma samples with known results regarding chromosomes 13, 18, 21, X and Y. They were sequenced in six different experiments, uploaded to NIPTviewer and assigned to a clinical laboratory geneticist for interpretation. The results of all previously analyzed samples were replicated.

**Conclusion:**

NIPTviewer facilitates NIPT results interpretation and has been implemented as part of a NIPT analysis routine that was accredited by the national accreditation body for Sweden (Swedac).

**Supplementary Information:**

The online version contains supplementary material available at 10.1186/s12920-025-02086-8.

## Background

Noninvasive prenatal testing (NIPT) is an efficient technique to screen for fetal chromosomal aneuploidies that are caused by the presence of an extra or missing copy of a chromosome. The method analyzes small fragments of cell-free DNA that are circulating in a pregnant woman’s blood (cfDNA). The DNA fragments arise when cells enter apoptosis, in which DNA is released into the bloodstream. Most cfDNA in maternal blood originates from the mother, with the fetal component (cffDNA) contributing 10–15% of the total cfDNA at 10–20 weeks of gestation (the most common time for NIPT) [1]. Analyzing cffDNA from peripheral maternal blood samples provides an opportunity for early detection of certain genetic abnormalities without an increased risk of miscarriage, which may follow traditional invasive sample collection (chorion villi biopsy or amniocentesis) [2]. The potential of NIPT in improving prenatal care has led to its implementation as a screening technique in many countries [3]. Still, unlike invasive tests, NIPT is not a diagnostic method for confirmation of trisomies in pregnancies. The presence of false positives and false negatives reported in studies using NIPT hinder its adoption as a definitive test for diagnosing trisomies [4]. NIPT is primarily used to detect the presence of additional chromosomes, in particular trisomy 21 (Down syndrome), trisomy 18 (Edwards syndrome), trisomy 13 (Patau syndrome) and an extra or missing copy of the sex chromosomes. In brief, the test quantifies the amount of cffDNA from each chromosome in the sample and estimates the ratio of amounts from test and reference chromosomes. A positive sample has a ratio that is different compared to the distribution in diploid samples. Typically, the ratio is higher due to the presence of more cffDNA from the additional chromosome, which indicates a trisomy.

Commercially available NIPT tests for use in diagnostic laboratories include DNA sequencing-based analyses, typically using shallow whole genome sequencing. One such commercial application is Illumina’s VeriSeq NIPT Solution v1 [5], which can produce NIPT results in two days. The test analyzes 16 samples per sequencing run and includes a proprietary software solution that provides output in a comma separated file (csv). The actual interpretation of the test results involves clustering experiment data with data from earlier runs to separate normal sample chromosomal ratios from samples with trisomies. With this approach, trisomies appear as outliers in a scatter plot. This visualization can be implemented using plot functions in any spreadsheet or statistical software, however such solutions typically give rise to multiple manual steps which may be both laboursome and introduce human errors, making the interpretation less reliable. Furthermore, spreadsheet solutions typically have little traceability support which is important in clinical laboratories. A more reliable solution is required to store data for multiple run comparisons and to track user activity. With these goals in mind we developed NIPTviewer, a web application that imports and validates the output from the NIPT analysis and visualizes the results by providing scatter plots and tables that compare the current analysis results to results from previous runs, simplifying the interpretation of the test results.

### Implementation

NIPTviewer is a web application developed in Python (3.8+) that uses Materialize (1.0.0) [6] to provide an appealing user interface. It utilizes Django (3.1.1) [7] as its web framework, providing essential functionalities such as user management and authentication. The application relies on a database for efficient data storage, with default support for databases like Sqlite, PostgreSQL, or Microsoft SQL. Furthermore, NIPTviewer can easily be configured to support other databases like Oracle, MariaDB or Mysql.

NIPTviewer utilizes pandas (1.5.3) [8] for data parsing, which enables easy processing and manipulation of data, and nvd3 (1.8.6) [9] to generate interactive charts for effective data visualization. To perform statistical calculations, NIPTviewer relies on SciPy (1.9) [10], which offers a comprehensive library of scientific and statistical functions.

The source code of NIPTviewer adheres to the PEP8 standard for improved code readability, ensured through the use of Pycodestyle (2.6.0) [11]. The Django test-execution framework is employed to rigorously test data parsing and function behavior, guaranteeing intended functionality.

**Fig. 1:**
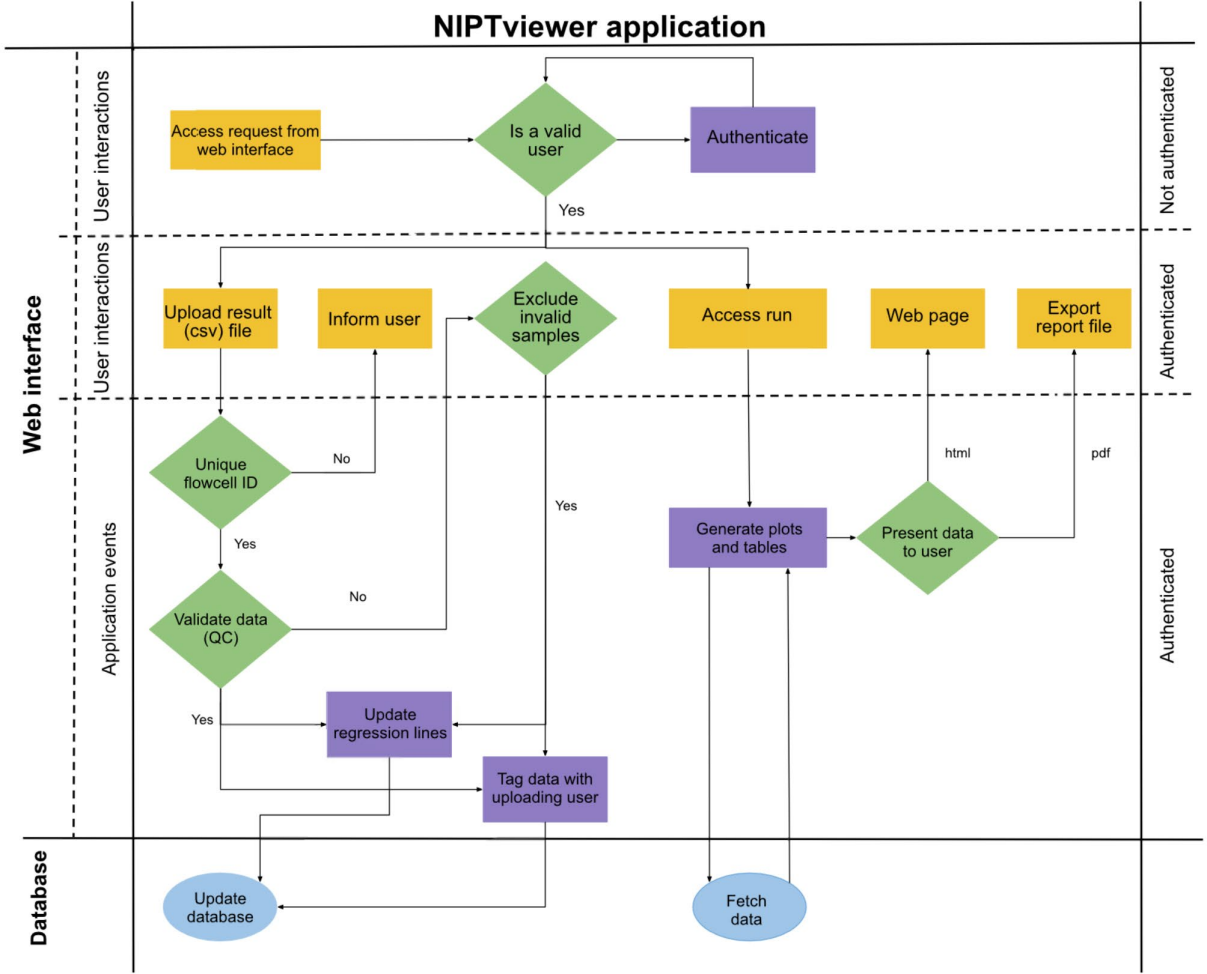
Schematic image of the NIPTviewer processing workflow illustrates the information flow and various operations within the application. NIPTviewer is composed of two components: a database responsible for storing and managing data and a web interface for user interactions

The data analysis processing workflow is designed to be straightforward and easy to use (Fig. 1). It starts when an authenticated user uploads the VeriSeq NIPT Analysis Software output .csv file to the application. During upload the result data is tagged with user information, making it possible to track which user performed the import. Test results are displayed using a combination of charts and tables, providing the user with a graphical overview of the data (Additional file 1). The charts are interactive and offer a visual representation of the data from the current experiment run, plotted over data from previous experiments. Tables display data from the current experiment run and highlight data points that deviate from the expected in a normal sample. When data has been inspected, the user has the option to export the visualizations as a .pdf report file that can be used in external systems for reporting and archiving purposes (see supporting information for examples of a NIPT report (Additional file 2) and a NIPT QC report (Additional file 3) .

### Deployment

Each release of the software is automatically packaged as a docker image and uploaded to dockerhub [12] where it is publicly accessible. To facilitate deployment, docker-compose and kubernetes configuration files exist in the github repository [13] where the source code is available under the MIT license. The application has been tested and runs on Firefox, Google Chrome, Microsoft Edge and Safari. Documentation, including installation instructions, is available at Read the Docs [14].

### Setup

NIPTviewer was developed using Django, which offers a high level of flexibility in the setup process and allow users to tailor the system according to their specific needs. To illustrate this we have deployed NIPTviewer using two different system setups: single server and multi-server. In the single server deployment, NIPTviewer and PostgreSQL were run on the same server but in separate Docker containers. This configuration allows efficient utilization of system resources while maintaining separation between the application and the database. In the multi-server setup, NIPTviewer was deployed in conjunction with Microsoft SQL on separate servers. This approach allows for distributed architecture, enabling scalability and improved performance by distributing the workload across multiple servers.

These different deployment options provide users the flexibility to choose what setup best suit their requirements, whether it is a compact and integrated setup or a distributed setup for enhanced performance and scalability.

### Usage

The supported import file format is the output .csv file that is generated by the Veriseq NIPT Analysis Software 16 Samples (1.4.0) [5]. The file name should begin with a date [YYMMDD]. Data in the file is used both to assess run and sample quality and to inform on sample aneuploidy status. The following metrics are of particular interest:

- Chromosome coverage distribution per sample - used to detect unusual patterns which could indicate processing problems or sample issues. It may also reflect actual chromosomal abnormalities.
- Fetal fraction (FF) - refers to the percent of cell-free circulating DNA in a maternal blood sample that is derived from the placenta. Uploaded data from the current experiment is displayed together with historical data from earlier experiments to enable the user to look for trends between runs.
- Normalized Chromosomal Denominator (NCD) values - are used to indicate chromosomal abnormalities with denominator chromosomes or processing errors. Uploaded data from the current experiment is displayed together with historical data from earlier experiments to enable the user to look for trends.
- Normalized chromosome values (NCV) - scaled to be equivalent to the commonly used Z-score [15]. Vales estimate how different a test result is compared to the average diploid ratio. Scatter plots with NCV chr13/18/21/X/Y data against FF (fetal fraction) are displayed together with historical data to identify samples that appear as outliers to the diploid sample cluster.

A plot is also generated for NCV X vs. NCV Y. This plot is particularly useful for identifying sex chromosome abnormalities.

Details on plots and metrics are available in the Additional file 1 and in the online documentation [14].

Data values from the analysis are available using the mouse-over feature in the graphs, or displayed in tables to give the user a quick overview of all values. In the tables, values above or below defined thresholds are highlighted in red, indicating they should be investigated more thoroughly during interpretation.

### Clinical implementation

A clinical competency test of 70 normal samples was performed as a first step to establish the Veriseq NIPT Analysis in our lab and to ensure that all parameters defined by the vendor were within their reference intervals. As a second step, with the certificate from the competency test, we performed a clinical verification with 84 plasma samples from singleton pregnancies that were sequenced in six separate runs. The verification included samples previously analyzed either by the Verify Prenatal test at Illumina Clinical Services Laboratory, US (*n* = 66) or by a reference laboratory at Turku University Hospital, Turku, Finland (*n* = 18). Both normal samples and samples with aneuploidies were included (five samples with trisomy 13, thirteen samples with trisomy 18, sixteen samples with trisomy 21, three samples with sex chromosome alterations and 47 normal samples). A maximum of three samples presenting with the same trisomy were included in the same run.

The verification samples were processed according to the guidelines of the manufacturer. In brief, libraries were constructed from cfDNA, quantified, diluted and pooled, followed by sequencing on a NextSeq550Dx (Illumina Inc, San Diego, CA). Sequence data was processed by the cADAS pipeline in the VeriSeq NIPT Analysis Software (Illumina) as implemented on a pre-installed and dedicated VeriSeq NIPT Analysis Server (Illumina). The analysis includes quality control steps, demultiplexing, mapping, coverage analysis and estimations of NCV and FF. The result file was imported into NIPTviewer followed by interpretation by a clinical laboratory geneticist. Data from the verification was used to determine the NCV threshold values and a regression line for NCV(X) vs. NCV(Y) (Additional file 1). After the verification, NIPTviewer was calibrated to receive clinical samples.

## Results

All criteria in the clinical verification were met, in particular the concentrations of the sequence libraries were 10-250nM, the cluster densities were 140–250 K/mm2, high quality sequence data was generated (Q30 > 95%), fetal fractions were ≥ 2% (range 2-23%) and all samples included in the verification replicated the previous results regarding chromosomes 13, 18, 21, X and Y. As a result, the analysis was approved for clinical routine use.

Based on the clinical verification data set, NCV threshold values could be confidently set for chromosomes 13, 18 and 21 to identify trisomies at NCV > 4 and fetal fraction ≥ 2%, with an inconclusive span at NCV 3–4. These threshold values are now implemented as default separator lines in the graphs of NIPTviewer (Additional file 1). The verification set was also used to establish a regression line with corresponding 99% confidence intervals (3 standard deviations of the mean) in the plot displaying normalized sex chromosome values (NCV(X) vs. NCV(Y)) (Supplementary Fig. 9, Additional file 1).

With the NCV(X) vs. NCV(Y) regression line and the NCV threshold values incorporated, NIPTviewer was implemented as part of a NIPT analysis routine that was accredited by the national accreditation body for Sweden (Swedac) and launched in clinical production in November 2020.

## Discussion

We developed NIPTviewer as a tool to facilitate clinical interpretation of NIPT results and to minimize manual data entry steps. The tool has been deployed in two different setups and is used by hospital staff to visualize NIPT analysis results and to guide the interpretation of the results. The visual inspection makes it easy to identify individual data points that deviate from the distributions of historical, mostly normal, samples. Furthermore, because samples with the same fetal chromosomal aneuploidies cluster, medical geneticists expect any sample with a trisomy to cluster with previously analyzed samples with the same variation. As a result, the visualizations potentiates a fast interpretation of test results, but also a means to identify inconclusive results whenever data points do not cluster as expected. NIPTviewer also provides traceability and minimizes manual steps that could introduce human errors. The first version of NIPTviewer was implemented in clinical production in November 2020 at Uppsala University Hospital and until April 2024 a total of 4941 samples were successfully uploaded to and visualized in NIPTviewer as part of the clinical NIPT analysis. The medical geneticists who have been working with NIPTviewer claim that the application is easy to work with, intuitive and fast. It simplifies and speeds up the interpretation process and presents them with all the information they need in order to be confident that test results are accurate.

## Conclusions

NIPTviewer provides a visualization of NIPT results from the VeriSeq NIPT Solution v1 that gives clinical staff a good overview of how individual data points cluster compared to historical data. The application include functionality for user management and authentication that are important for traceability and can output PDF reports that may be used in the clinical reporting process. Deployment options provide laboratories the flexibility to choose what setup best suit their requirements.

## Electronic supplementary material

Below is the link to the electronic supplementary material.

**Supplementary Material 1**: **Additional file 1**

**Supplementary Material 2**: **Additional file 2**

**Supplementary Material 3**: **Additional file 3**

## Data Availability

No datasets were generated or analysed during the current study.

## Availability and requirements

Project name: NIPTviewer

Project home page: https://github.com/clinical-genomics-uppsala/NIPTviewer

Programming language: Python (3.8+), Django (3.1.1)

Other requirements: See the Implementation section

License: MIT

Any restrictions to use by non-academics: No

## Abbreviations

NIPT: Noninvasive prenatal testing
cfDNA: Cell-free DNA
cffDNA: Cell-free fetal DNA
FF: Fetal fraction
NCD: Normalized chromosomal denominator
NCV: Normalized chromosomal value
