# Supplementary material for: Visualization using NIPTviewer support the clinical interpretation of noninvasive prenatal testing results
Source: BMC Med Genomics. 2025 Jan 20;18:15. doi: 10.1186/s12920-025-02086-8 (PMC11748546; doi:10.1186/s12920-025-02086-8)
Supplement: Supplementary file 1 — Supplementary Material 1: Additional file 1 [file 12920_2025_2086_MOESM1_ESM.pdf]

Visualization using NIPTviewer to support the clinical interpretation of Noninvasive Prenatal Testing results

Supplementary information

Content

On normalized chromosome value (NCV) ranges 1

Information about plots and tables included in the visualization. 2

Supplementary table 1. Overview table 2

Supplementary table 2. NCV(13) per sample table 2

Supplementary figure 1. Read distribution 3

Supplementary figure 2. Trend of NCV values 3

Supplementary figure 3. Trend of FF values 4

Supplementary figure 4. Per sample normalized coverage 4

Supplementary figure 5. NCV(13) per sample 5

Supplementary figure 7. NCV(21) per sample 6

Supplementary figure 8. NCV(X) and NCV(Y) explained 7

Supplementary table 3. Sex chromosome NCV ranges 7

Supplementary figure 9: NCV(X) vs NCV(Y) plot 8

On normalized chromosome value (NCV) ranges

Interpretation thresholds (NCV-values) to identify trisomies could be confidently set for chromosomes 13, 18 and 21 at NCV >4 and fetal fraction ≥2%, with an inconclusive span at NCV 3-4. Sex chromosome interpretation posed a considerable challenge however, and correct classification was dependent on previously tested samples. NCV-value ranges could be confidently defined for XX (NCV(X) -3 to 3, NCV(Y) <3), X0 (NCV(X) <-4, NCV(Y) <3) and XXX (NCV(X) >4, NCV(Y) <3) (supplementary table 3). Because the a priori assumption in Veriseq NIPT v1 solution is that the pregnant woman is diploid, mosaic X0 and XXX women could not be assessed, hence, this was a limitation. Regarding NCV(Y) we only defined the threshold for NCV(X) and NCV(Y) for normal male sex chromosomes, XY (NCV(X) <-4, NCV(Y) >4), hence XYY and XXY could only be indicated based on proximity clustering of previous samples (supplementary figure 8 and supplementary figure 9). The negative predictive value, NPV, for women of all ages, of XX and XY was estimated to >99%. The positive predictive value, PPV, for sex chromosome abnormalities was however estimated to <50% for women at 30 years of age (X0 41%, XXX 27%, XXY 29%, XYY 25%). Therefore, we decided to only recommend sex chromosome assessment when there was a clinical indication, for example X-chromosome disease in the family, with the purpose to determine if invasive diagnostics is necessary based on the gender of the fetus. If the fetus presented with ultrasound abnormalities, invasive diagnostics was recommended.

Information about plots and tables included in the visualization.

For more information, please visit documentation at https://niptviewer.readthedocs.io.

Supplementary table 1. Overview table

Overview table

Run data

Sample	NCV 13	NCV 18	NCV 21	NCV X	NCV Y	Fetal fraction
sample-low-ff	0.019	-0.359	-0.119	-1.506	41.876	1.0%
sample13-1	20.023	-0.050	-2.886	-2.930	-0.364	9.0%
sample13-2	17.837	0.145	0.080	1.144	-0.989	7.0%
sample18-1	0.819	10.432	0.745	0.967	-1.668	6.0%
sample18-2	-0.507	17.094	1.366	0.492	-0.392	6.0%
sample21-1	0.178	0.585	22.409	-0.851	0.183	11.0%
sample21-2	-0.674	0.341	24.444	-12.966	212.854	12.0%
sampleX-1	0.979	0.282	-0.132	-5.790	107.888	7.0%
sampleX-2	-0.135	-0.927	-1.130	-11.503	158.713	11.0%
sampleY-1	-0.112	-0.158	-0.559	-7.633	117.909	8.0%
sampleY-2	-0.180	-0.136	-1.544	-20.101	312.135	21.0%
XY-XX-lot5	0.475	1.101	-0.502	-3.751	61.012	9.0%

Supplementary table 1. Table showing the values from the input data file. Values that deviate from expected ranges are highlighted to guide the attention of the person examining the results.

Supplementary table 2. Table of NCV(13) per sample.

Per metric table

Sample	NCV 13	Fetal fraction
sample-low-ff	0.019	1.0%
sample13-1	20.023	9.0%
sample13-2	17.837	7.0%
sample18-1	0.819	6.0%
sample18-2	-0.507	6.0%
sample21-1	0.178	11.0%
sample21-2	-0.674	12.0%
sampleX-1	0.979	7.0%
sampleX-2	-0.135	11.0%
sampleY-1	-0.112	8.0%
sampleY-2	-0.180	21.0%
XY-XX-lot5	0.475	9.0%

Supplementary table 2. Table showing the exact values of NCV for individual chromosomes (in this case NCV(13) and the fetal fraction from the input data file. Values that deviate from expected ranges are highlighted to guide the attention of the person that is examining the results.

Supplementary figure 1. Read distribution

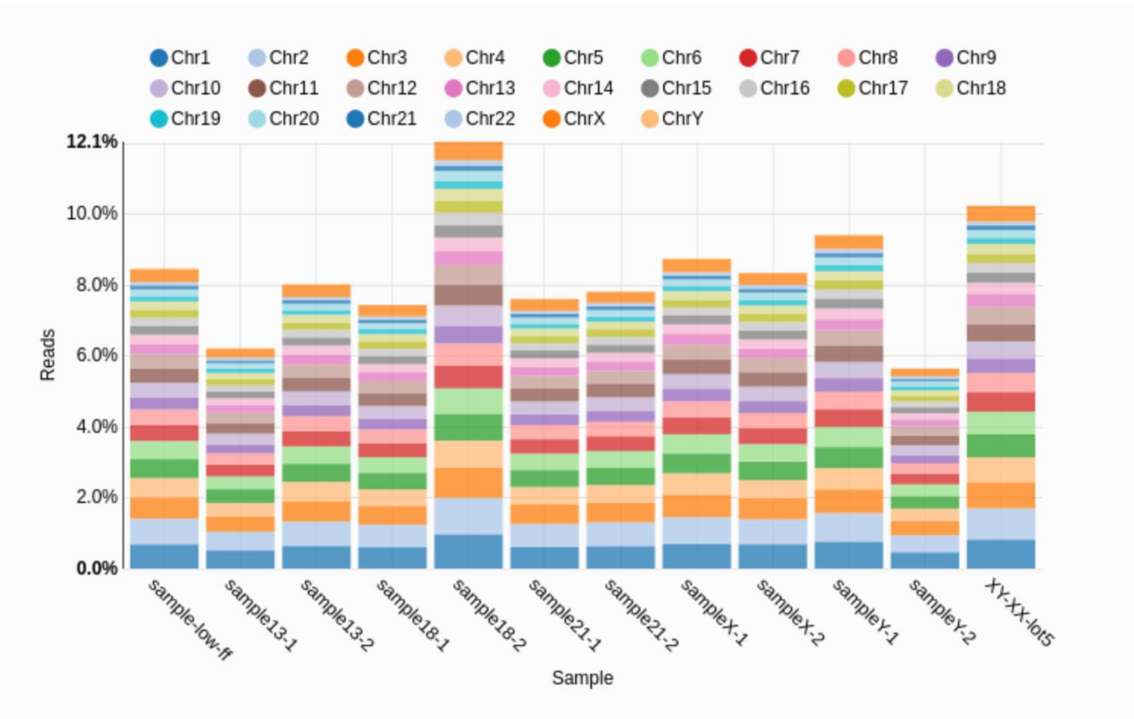

Supplementary figure 1. The plot shows the distribution of reads per chromosome in each sample that were analyzed in the same sequencing experiment.

Supplementary figure 2. Trend of NCV values

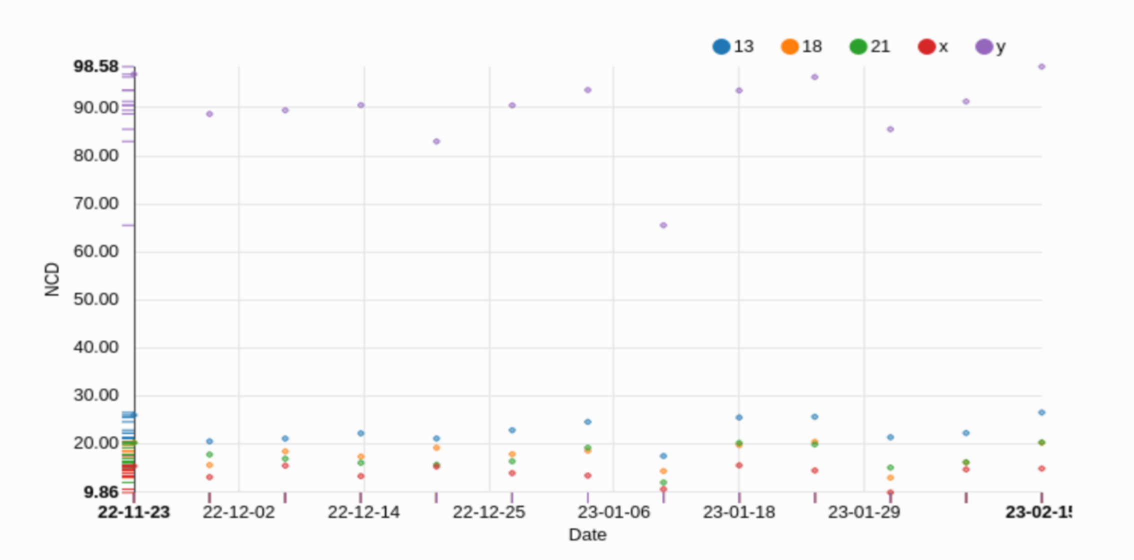

Supplementary figure 2. The five NCV values from multiple sequencing experiments are displayed on a time axis. Deviations may suggest problems with a sequencing experiment.

Supplementary figure 3. Trend of FF values

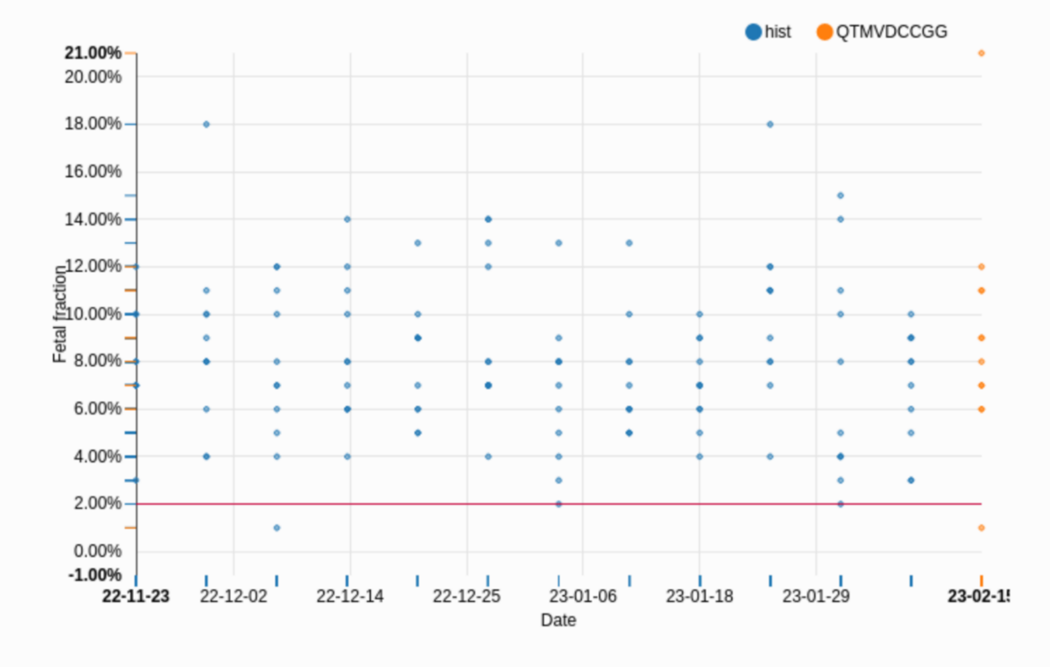

Supplementary figure 3. The fetal fraction (FF) values from multiple sequencing experiments are displayed on a time axis. Deviations may suggest problems with individual samples or with a sequencing experiment.

Supplementary figure 4. Per sample normalized coverage

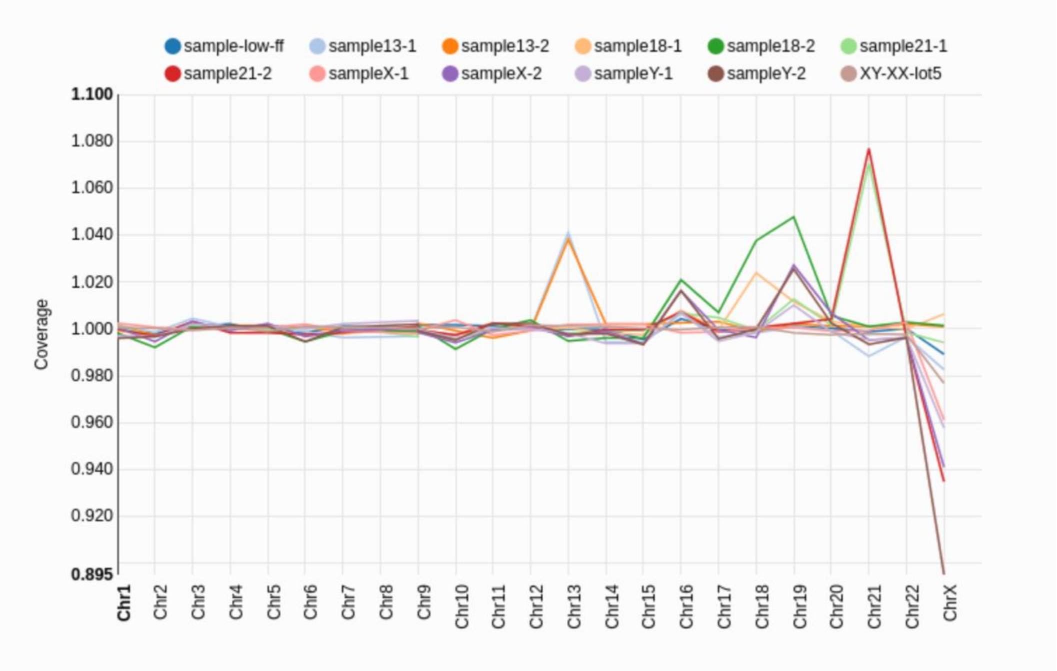

Supplementary figure 4. Per sample normalized coverage may point to chromosomal aberrations that could influence the NCV value calculations. Please note the normalized coverage change in samples with trisomy 13, 18 and 21 in this dataset. There is also a wide distribution of normalized coverage values for the X chromosome due to gender.

Supplementary figure 5. NCV(13) per sample

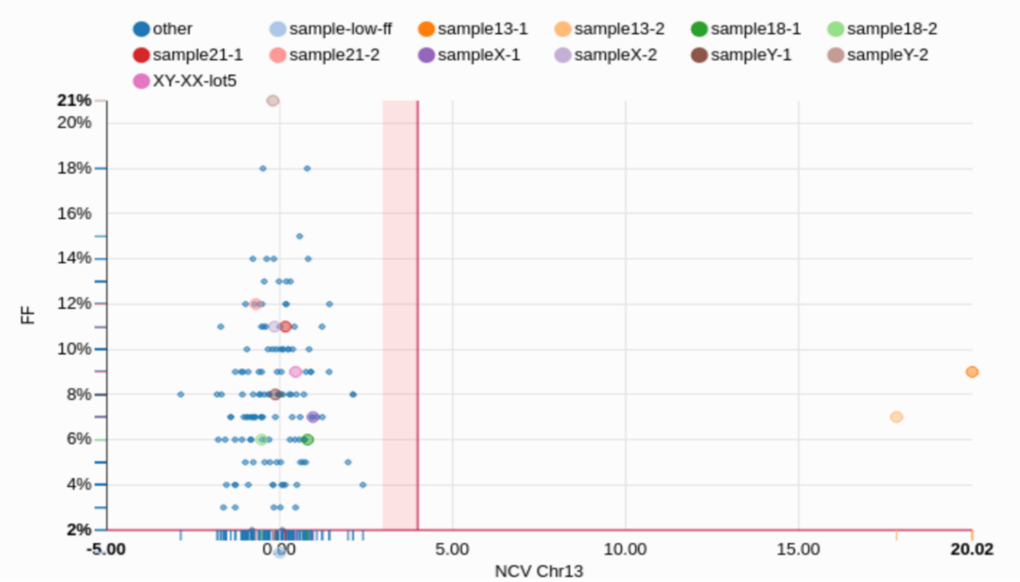

Supplementary figure 5. Per sample NCV(13) values are displayed on the x-axes with FF values on the y-axes. Data points from the current sequencing experiment are displayed in colors as indicated by the legend. In the NIPTviewer application the mouse-over feature can be used to show the exact value of a data point. Data points from earlier sequencing experiments are plotted with smaller blue dots to show how the current data points compare to data from previous experiments. The line at NCV(13)=4 corresponds to the threshold that was defined by the verification experiments. Values between 3 and 4 are considered inconclusive.

Supplementary figure 6. NCV(18) per sample

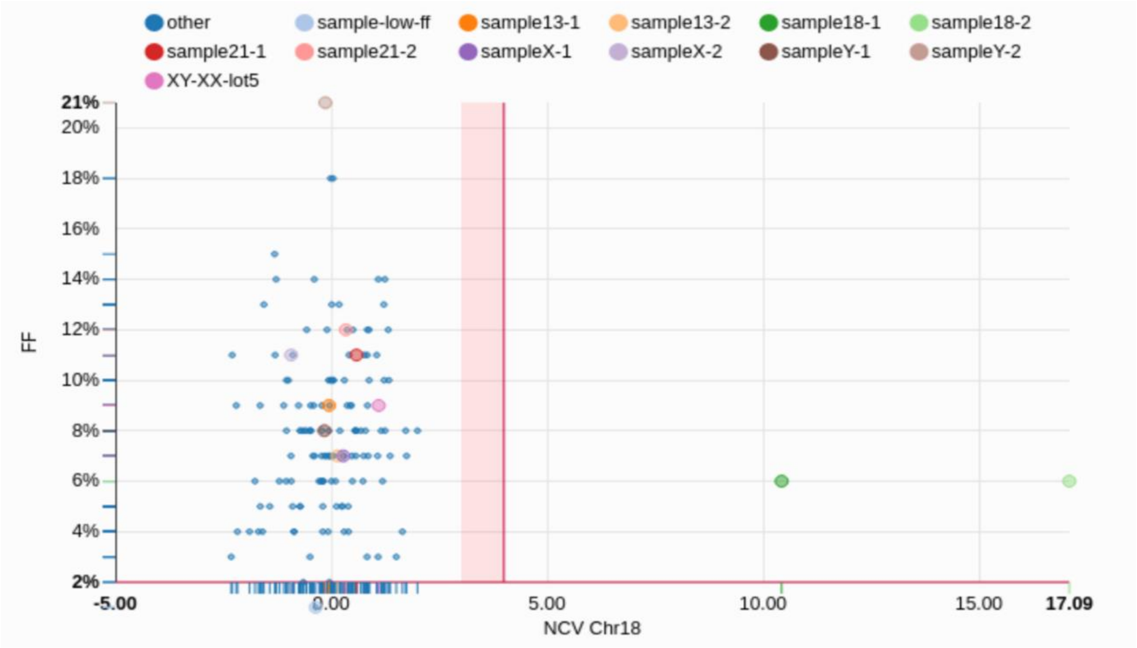

Supplementary figure 6. Per sample NCV(18) values are displayed on the x-axes with FF values on the y-axes. Data points from the current sequencing experiment are displayed in colors as indicated by the legend. In the NIPTviewer application the mouse-over feature can be used to show the exact value of a data point. Data points from earlier sequencing experiments are plotted with smaller blue dots to show how the current data points compare to data from previous experiments. The line at NCV(18)=4 corresponds to the threshold that was defined by the verification experiments. Values between 3 and 4 are considered inconclusive.

Supplementary figure 7. NCV(21) per sample

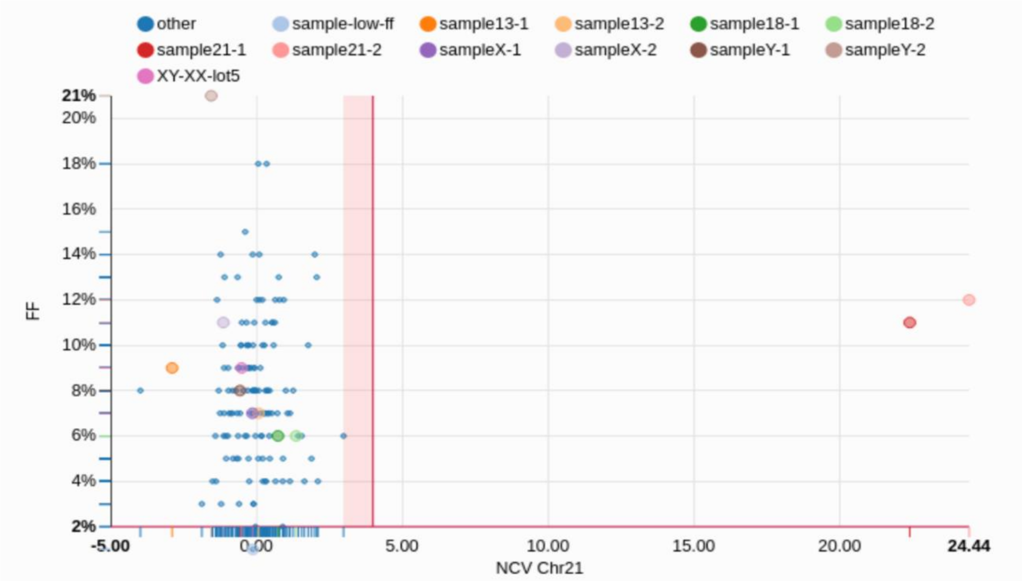

Supplementary figure 7. Per sample NCV(21) values are displayed on the x-axes with FF values on the y-axes. Data points from the current sequencing experiment are displayed in colors as indicated by

the legend. In the NIPTviewer application the mouse-over feature can be used to show the exact value of a data point. Data points from earlier sequencing experiments are plotted with smaller blue dots to show how the current data points compare to data from previous experiments. The line at NCV(21)=4 corresponds to the threshold that was defined by the verification experiments. Values between 3 and 4 are considered inconclusive.

Supplementary figure 8. NCV(X) and NCV(Y) explained

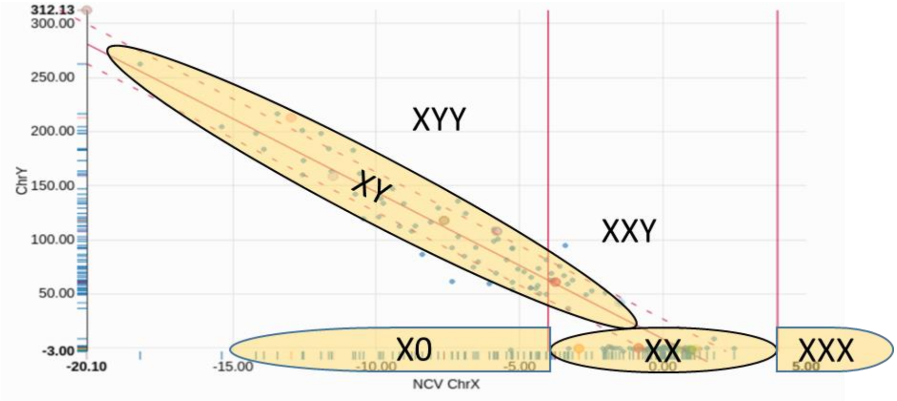

Supplementary figure 8. Expected cluster distributions of sex chromosome NCV values across the different sex chromosome groups. Individuals without a Y chromosome are expected to have NCV(Y)<3. Suggestive thresholds between clusters are given in supplementary table 3.

Supplementary table 3. Sex chromosome NCV ranges

Sex chromosome setup	*NCV(X)*	*NCV(Y)*
XX	-3 < X < 3	<3
X0	<-4	<3
XXX	> 4	<3
XY	<-4	>4
Inconclusive, (probably XX)	3-4	<3
Inconclusive, (probably XY)	3-4	>4

Supplementary table 3. Expected normalized chromosome values (NCV) of the sex chromosomes at fetal fraction values ≥2%. Sex chromosome trisomy (XXX, XXY or XYY) is suspected if sample data does not cluster with the XY or XX clusters (supplementary figure 8).

Supplementary figure 9: NCV(X) vs NCV(Y) plot

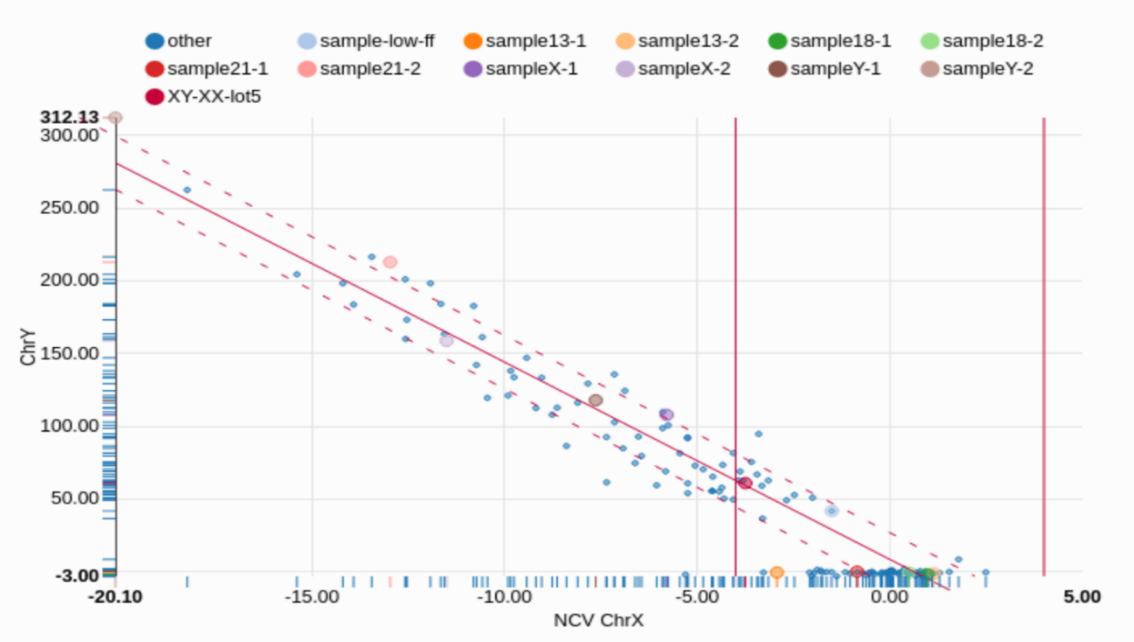

Supplementary figure 9. Sex chromosome NCV values. Vertical lines correspond to threshold values NCV(X) = -4 and NCV(X) = 4, respectively. Included in the visualization is also a regression line with corresponding 99% confidence intervals (3 standard deviations of the mean) of NCV(Y) vs NCV(X) in individuals with at least one Y chromosome.
