# Supplementary material for: Visualization using NIPTviewer support the clinical interpretation of noninvasive prenatal testing results
Source: BMC Med Genomics. 2025 Jan 20;18:15. doi: 10.1186/s12920-025-02086-8 (PMC11748546; doi:10.1186/s12920-025-02086-8)
Supplement: Supplementary file 2 — Supplementary Material 2: Additional file 2 [file 12920_2025_2086_MOESM2_ESM.pdf]

v1.10.0

NIPT Report

Clinical Genomics Uppsala

Flowcell: QTMVDCCGG
Run Data: 2024-08-28
Uploaded by: Johan Jansson (2024-08-28)
Status: PASS

Sample	NCV 13	NCV 18	NCV 21	NCV X	NCV Y	Fetal fraction
sample-low-ff	0.019	-0.359	-0.119	-1.506	41.876	1.0%
sample13-1	20.023	-0.050	-2.886	-2.930	-0.364	9.0%
sample13-2	17.837	0.145	0.080	1.144	-0.989	7.0%
sample18-1	0.819	10.432	0.745	0.967	-1.668	6.0%
sample18-2	-0.507	17.094	1.366	0.492	-0.392	6.0%
sample21-1	0.178	0.585	22.409	-0.851	0.183	11.0%
sample21-2	-0.674	0.341	24.444	-12.966	212.854	12.0%
sampleX-1	0.979	0.282	-0.132	-5.790	107.888	7.0%
sampleX-2	-0.135	-0.927	-1.130	-11.503	158.713	11.0%
sampleY-1	-0.112	-0.158	-0.559	-7.633	117.909	8.0%
sampleY-2	-0.180	-0.136	-1.544	-20.101	312.135	21.0%
XY-XX-lot5	0.475	1.101	-0.502	-3.751	61.012	9.0%

Clinical Genomics Uppsala

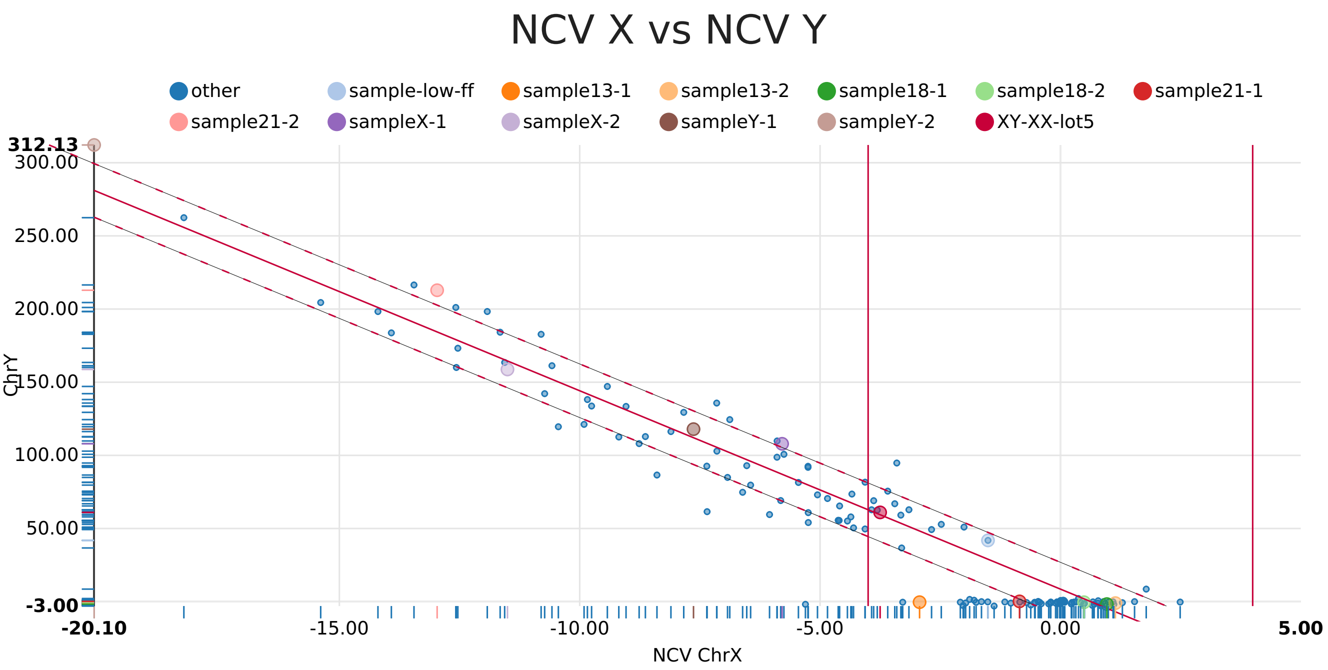

Sample	NCV X	NCV Y
sample-low-ff	-1.506	41.876
sample13-1	-2.930	-0.364
sample13-2	1.144	-0.989
sample18-1	0.967	-1.668
sample18-2	0.492	-0.392
sample21-1	-0.851	0.183
sample21-2	-12.966	212.854
sampleX-1	-5.790	107.888
sampleX-2	-11.503	158.713
sampleY-1	-7.633	117.909
sampleY-2	-20.101	312.135
XY-XX-lot5	-3.751	61.012

Clinical Genomics Uppsala

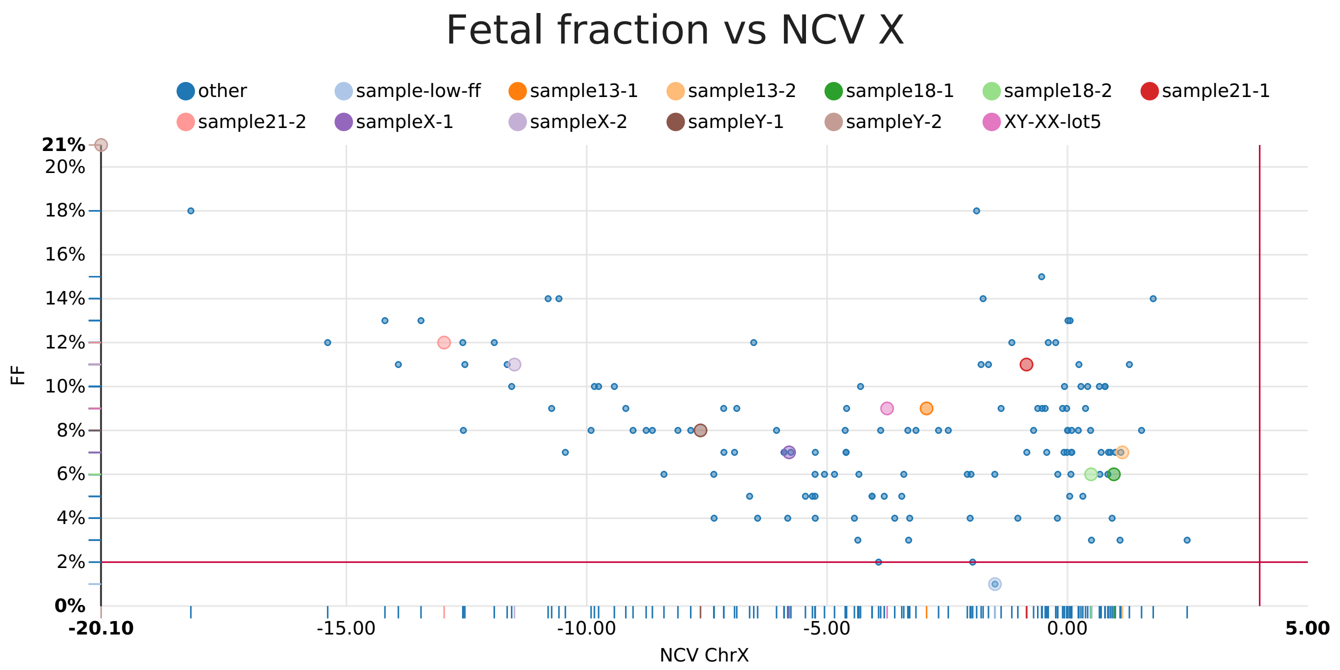

Sample	NCV X	Fetal fraction
sample-low-ff	-1.506	1.0%
sample13-1	-2.930	9.0%
sample13-2	1.144	7.0%
sample18-1	0.967	6.0%
sample18-2	0.492	6.0%
sample21-1	-0.851	11.0%
sample21-2	-12.966	12.0%
sampleX-1	-5.790	7.0%
sampleX-2	-11.503	11.0%
sampleY-1	-7.633	8.0%
sampleY-2	-20.101	21.0%
XY-XX-lot5	-3.751	9.0%

Clinical Genomics Uppsala

Fetal fraction vs NCV Y

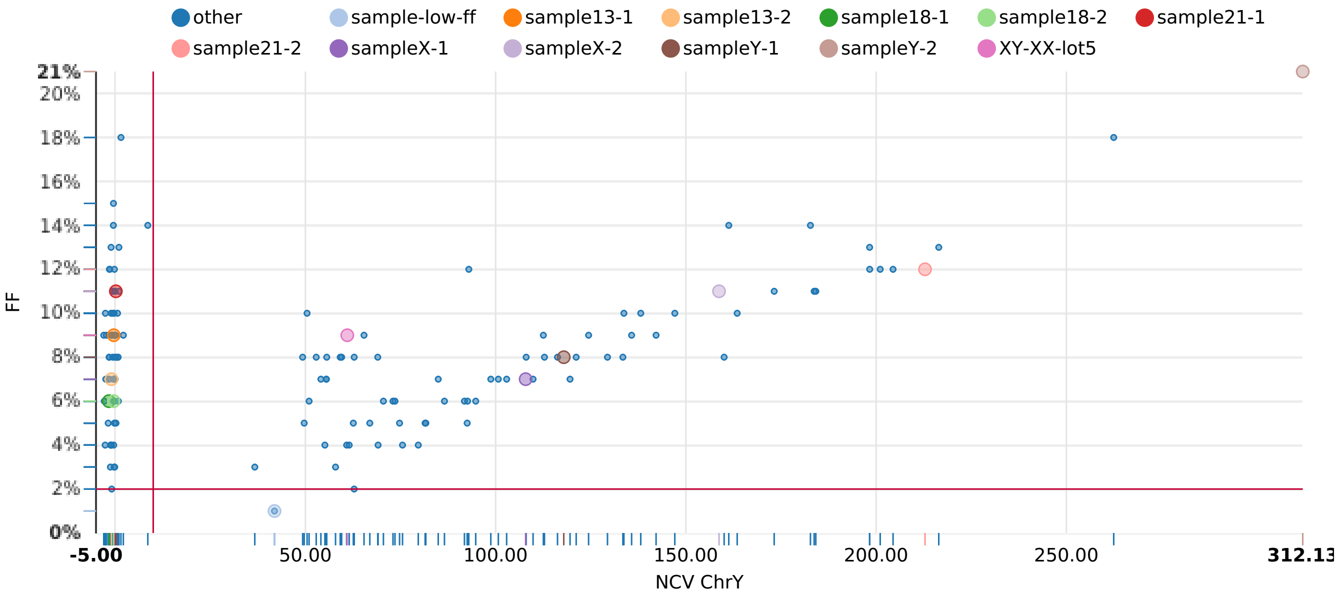

Sample	NCV Y	Fetal fraction
sample-low-ff	41.876	1.0%
sample13-1	-0.364	9.0%
sample13-2	-0.989	7.0%
sample18-1	-1.668	6.0%
sample18-2	-0.392	6.0%
sample21-1	0.183	11.0%
sample21-2	212.854	12.0%
sampleX-1	107.888	7.0%
sampleX-2	158.713	11.0%
sampleY-1	117.909	8.0%
sampleY-2	312.135	21.0%
XY-XX-lot5	61.012	9.0%

Fetal fraction vs NCV 13

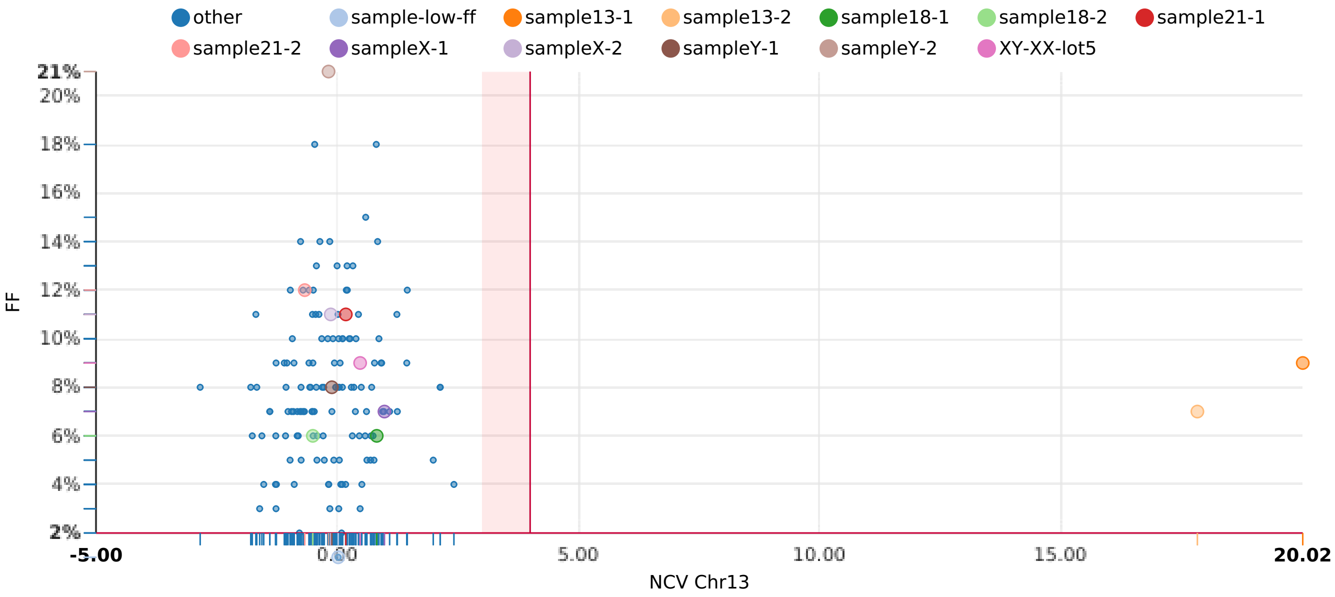

Sample	NCV 13	Fetal fraction
sample-low-ff	0.019	1.0%
sample13-1	20.023	9.0%
sample13-2	17.837	7.0%
sample18-1	0.819	6.0%
sample18-2	-0.507	6.0%
sample21-1	0.178	11.0%
sample21-2	-0.674	12.0%
sampleX-1	0.979	7.0%
sampleX-2	-0.135	11.0%
sampleY-1	-0.112	8.0%
sampleY-2	-0.180	21.0%
XY-XX-lot5	0.475	9.0%

Clinical Genomics Uppsala

Fetal fraction vs NCV 18

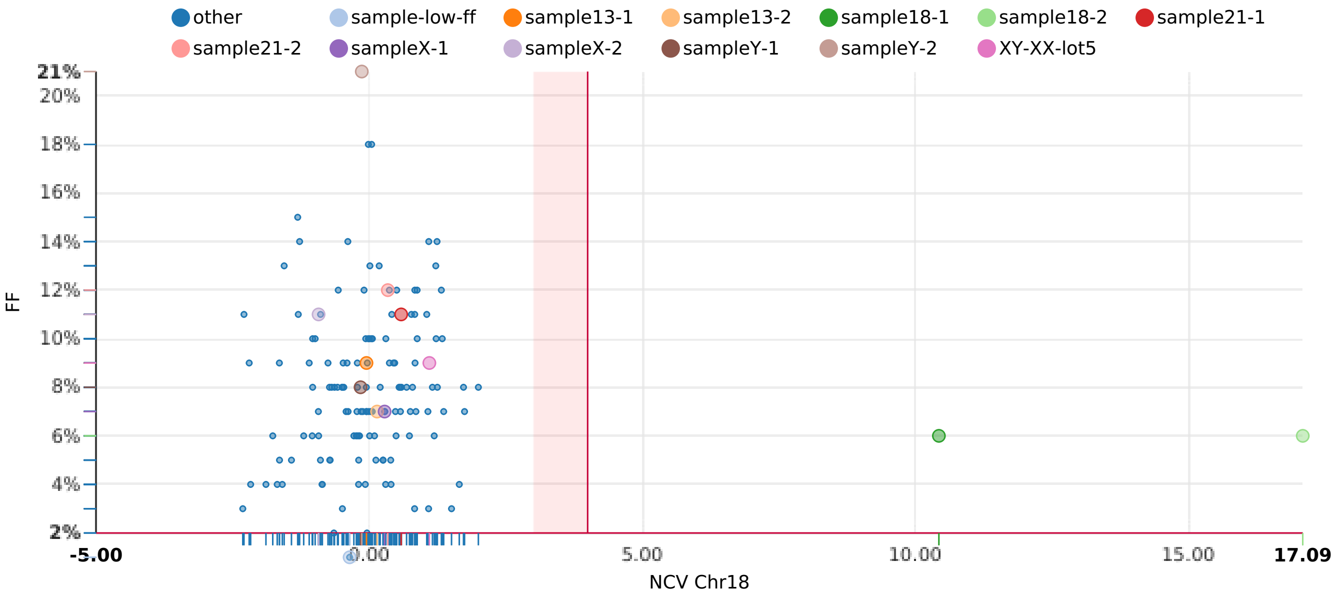

Sample	NCV 18	Fetal fraction
sample-low-ff	-0.359	1.0%
sample13-1	-0.050	9.0%
sample13-2	0.145	7.0%
sample18-1	10.432	6.0%
sample18-2	17.094	6.0%
sample21-1	0.585	11.0%
sample21-2	0.341	12.0%
sampleX-1	0.282	7.0%
sampleX-2	-0.927	11.0%
sampleY-1	-0.158	8.0%
sampleY-2	-0.136	21.0%
XY-XX-lot5	1.101	9.0%

Fetal fraction vs NCV 21

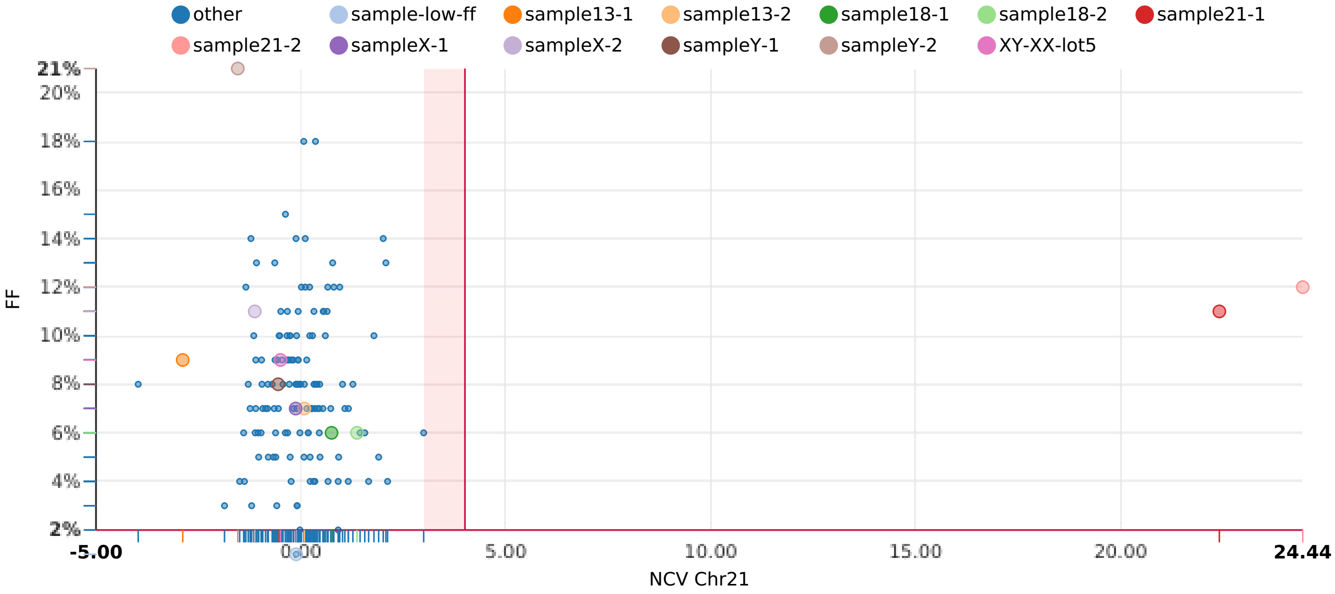

Sample	NCV 21	Fetal fraction
sample-low-ff	-0.119	1.0%
sample13-1	-2.886	9.0%
sample13-2	0.080	7.0%
sample18-1	0.745	6.0%
sample18-2	1.366	6.0%
sample21-1	22.409	11.0%
sample21-2	24.444	12.0%
sampleX-1	-0.132	7.0%
sampleX-2	-1.130	11.0%
sampleY-1	-0.559	8.0%
sampleY-2	-1.544	21.0%
XY-XX-lot5	-0.502	9.0%
