# Supplementary figures and images for: Visualization using NIPTviewer support the clinical interpretation of noninvasive prenatal testing results
Source: BMC Med Genomics. 2025 Jan 20;18:15. doi: 10.1186/s12920-025-02086-8 (PMC11748546; doi:10.1186/s12920-025-02086-8)

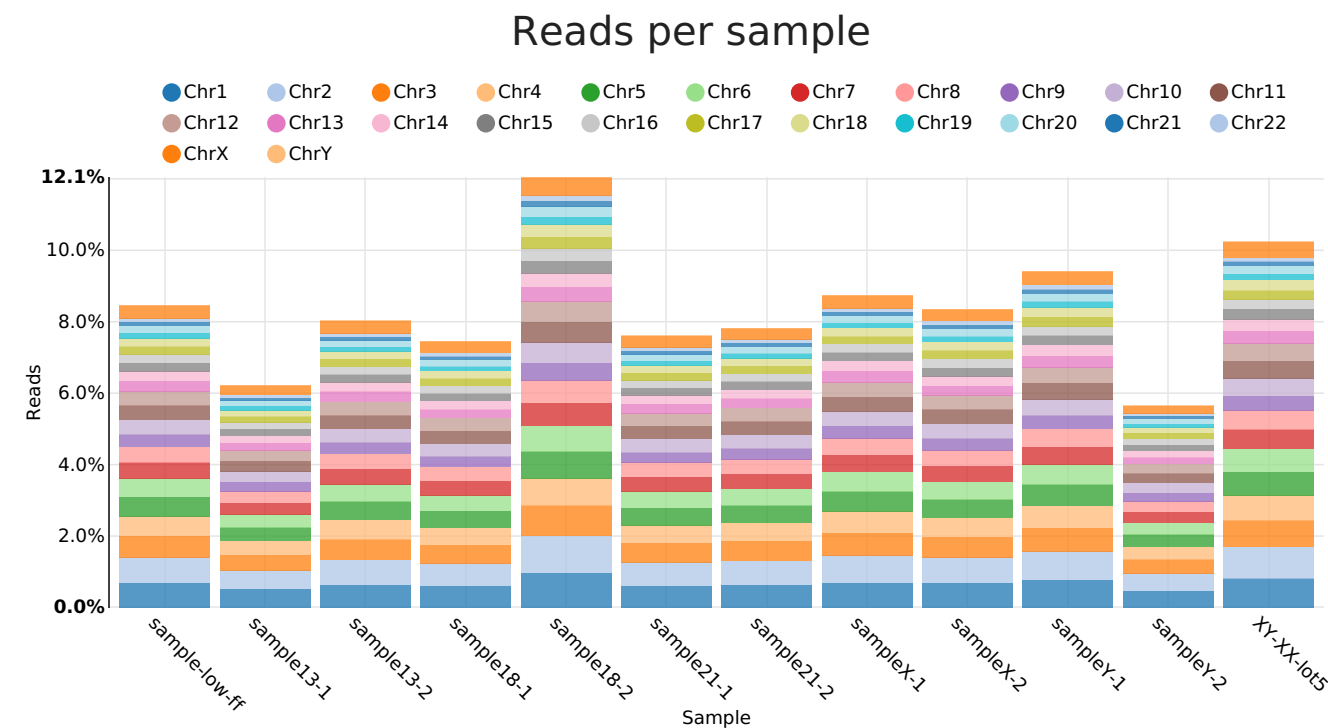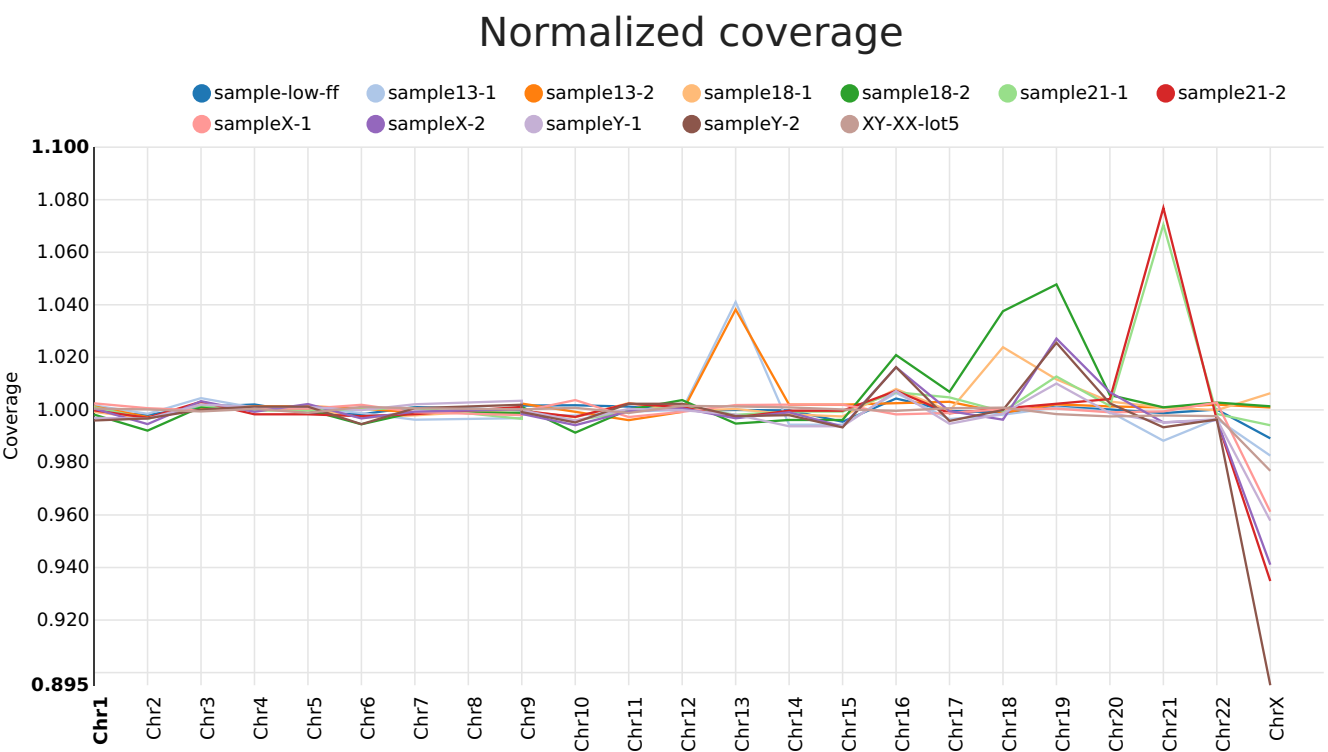

Fetal fraction

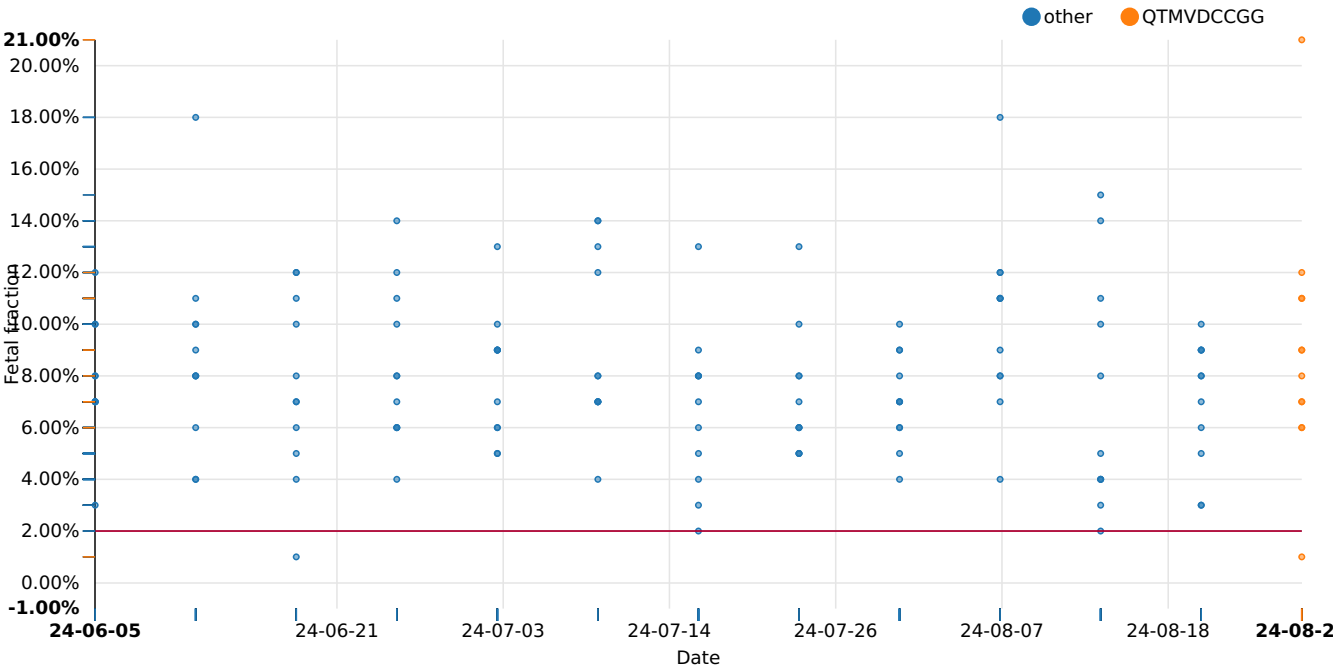

NCD

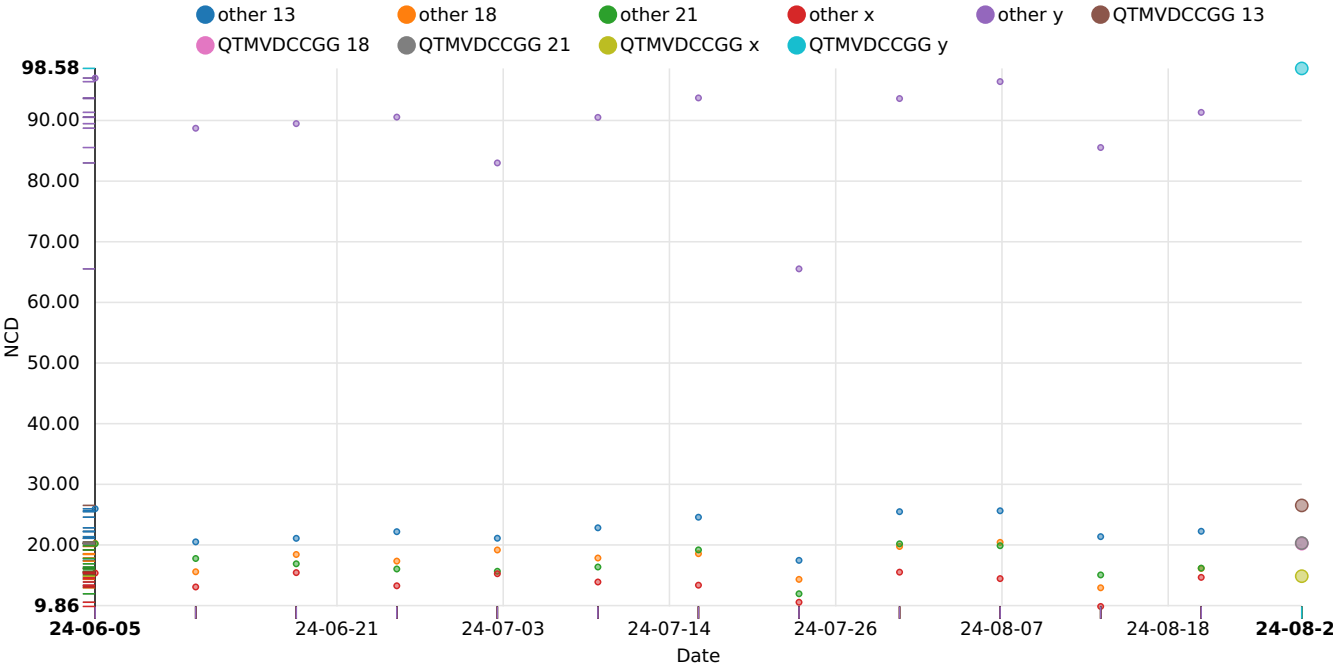

Supplement: Supplementary file 3 — Supplementary Material 3: Additional file 3 [file 12920_2025_2086_MOESM3_ESM.pdf]
